# Perspectives on Biological Monitoring in Environmental Health Research: A Focus Group Study in a Native American Community

**DOI:** 10.3390/ijerph15061129

**Published:** 2018-05-31

**Authors:** Melissa Gonzales, Elanda King, Jeanette Bobelu, Donica M. Ghahate, Teresa Madrid, Sheri Lesansee, Vallabh Shah

**Affiliations:** 1 Department of Internal Medicine, School of Medicine, University of New Mexico Health Sciences Center, MSC10 5550, Albuquerque, NM 87131, USA; enking814@gmail.com; 2 Department of Biochemistry and Molecular Biology, School of Medicine, University of New Mexico Health Sciences Center, MSC 08 4670 Albuquerque, NM 87131, USA; jbobelu@salud.unm.edu (J.B.); dneha@salud.unm.edu (D.M.G.); 3 Office for Diversity, University of New Mexico Health Sciences Center, MSC 09 5235 Albuquerque, NM 87131, USA; tmmadrid@salud.unm.edu; 4 Robert Wood Johnson Foundation Center for Health Policy, University of New Mexico, MSC 02 1645, Albuquerque, NM 87131, USA; sheri@unm.edu

**Keywords:** Native American, environmental exposure, biological monitoring, rural populations, focus group

## Abstract

*Background:* Reliance on natural resources brings Native American communities into frequent contact with environmental media, which, if contaminated, represents an exposure route for environmental pollutants. Native American communities vary in their perspectives on research and relatively little is known about the range of perspectives regarding the use of biological samples for environmental exposure assessment. *Methods:* Thirty-one members of Zuni Pueblo (median age = 40.0 years, range = 26–59 years) participated a series of four focus groups. Qualitative themes emerging from the focus group discussion transcripts were identified by content analysis. *Results*: Emergent themes included adequate informed consent, traditional beliefs, and personal choice. *Conclusions:* The discussions reinforced the central role of traditional values in the decision to participate in research involving biological samples for environmental exposure assessment. Decision-making required a balance between the perceived value of the proposed project and its purpose, with cultural perspectives surrounding the biological sample requested. We examine the potential for study bias and include recommendations to aid in the collaborative identification and control of unintended risks posed by the use of biological samples in environmental health studies in native communities.

## 1. Introduction

Native American communities, many of which are located in rural areas, rely on local natural resources for many daily aspects of their unique traditional lifestyles, community activities, occupations, and customs [1]. This reliance on natural resources brings community members into frequent contact with environmental media, which, if contaminated, represents an exposure route for environmental pollutants. As a result, Native American communities may be at increased risk for environmentally induced diseases [1]. The extraction of naturally occurring metals has significantly affected tribal lands, posing an elevated risk for metals exposure as a result of environmental releases. The mined regions are contaminated with arsenic, copper, uranium, and other heavy metals as well as remaining radionuclides, representing an exposure risk to metals associated with adverse health and developmental effects [2]. Harmon et al. (2017 and 2018) reported an association between arsenic and uranium from water intake and residential proximity to abandoned uranium mining sites with markers of pre-clinical cardiovascular effects [3,4]. In a recent review, Gonzales et al. (2018) identified Native American and other rural populations in the Southwest, Mountain West, and adjacent regions of the U.S. to be at risk for sufficient exposure to environmental metals to result in elevated risks for numerous adverse health outcomes [5]. Specifically, environmental exposure to arsenic, uranium, and cadmium were associated with increased incidence of diabetes, cardiovascular, and kidney disease among Native Americans in the region participating in the Strong Heart Study [6,7,8,9,10].

To assess the potential health risks posed by these pollutants, exposure may be quantified through measurement of pollutants or their metabolites in biological samples such as blood, urine, hair, or nails. This process is a more direct method of quantifying exposure than measurements in environmental media because it is a measure of the body burden or internal dose of the pollutant [11,12]. However, Native American communities vary in their perspectives on research in general, and relatively little is known about the range of perspectives among Native American communities regarding the use of biological samples for environmental exposure assessment [13,14,15]. Additionally, research in Native American communities is impacted by historically negative experiences with research and researchers as well as a heightened sensitivity to confidentiality, which may be amplified by rural and cultural factors [14,15]. In recognition of the potential ethical challenges of conducting biological monitoring studies among racial/ethnic minority populations and rural populations, the National Research Council has called for additional research on new approaches to address these challenges [16]. The U.S. Environmental Protection Agency has called for additional research on assessment and reduction of risk in susceptible populations to meet the needs of Native American tribes [1].

Our focus group study addresses the call to gain insights into the perspectives of a Native American community regarding the use of biological samples for environmental exposure assessment. Our specific objective was to gather data about the views of this community regarding the use of urine, blood, hair, and nail clippings to quantify exposure in environmental health research.

## 2. Materials and Methods

### 2.1. Recruitment and Participants

Eligible participants were adult members of Zuni Pueblo, New Mexico (NM) who lived on tribal lands located in the southwestern United States. The recruitment strategy included the use of in-person contact with potential participants and by posting informational flyers at local businesses and tribal programs in the Zuni community. Forty subjects were recruited to attend one of four focus groups held on Zuni Pueblo. Thirty-one individuals (62%) participated in a focus group (15 males, 16 females, median age = 40.0 years, age range = 26–59 years). All subjects gave their informed consent for inclusion before they participated in the study. The study was conducted in accordance with the Declaration of Helsinki, and the protocol was approved by the Human Research Protections Office of the University of New Mexico Health Sciences Center (IRB # 03-409, 2003) as well as the Indian Health Services Institutional Review Board. The Pueblo of Zuni tribal governance also approved the study protocol.

### 2.2. Focus Group Process

In a manner similar to talking circles used by many Native American communities, the focus group process permits each person to speak freely and without interruption [17]. Each speaker is expected to treat all other speakers with respect and to acknowledge and build on each contribution to produce an increasingly rich accumulation of thought [17]. Focus groups are an ideal method to obtain the perspectives of individual community members in the broader context of the community belief system.

The focus groups were conducted with the assistance of a facilitator and two scribes, all of whom were Zuni and fluent in Shiwi, the Zuni language, as well as in English. All focus group participants were proficient in English and the participants spoke only English during the focus group discussions. The scribes took notes and made audio recordings of the sessions. The facilitator transcribed the recordings. Names were not transcribed from the focus group recordings or into the written notes. After the transcriptions were compared to the audio recordings and notes for accuracy, the recordings were erased.

The focus group guide consisted of questions concerning the collection of biological samples, specifically blood, urine, hair, and nail clippings, in a hypothetical environmental health study. To introduce the concept of using biological samples to quantify the body burden of environmental exposure, participants were given several illustrative examples of biological samples and how the samples could be used in research to correlate environmental exposure with disease. Next, a hypothetical research study was proposed, in which the investigators hypothesized a relationship between environmental metals exposure and disease in the study community. The participants were then asked the reasons they and others in the community may or may not want to provide a biological sample (e.g., blood, urine, hair, or nail clippings) to evaluate the study hypothesis.

### 2.3. Content Analysis

A content analysis of the focus group data was conducted using an iterative process. The first step involved a review of the transcripts to identify qualitative themes that emerged from the data. Both the authors and the focus group facilitator, who was a member of the tribe, independently reviewed and coded the transcripts. Final thematic constructs were identified by comparison of the coding utilized by all reviewers.

## 3. Results

The goal of the focus groups was to identify the perspectives of tribal community members surrounding the collection of biological samples for environmental health research. On the basis of our content analysis of the focus group transcripts, three categories were identified: adequate informed consent, traditional beliefs, and personal choice.

### 3.1. Adequate Informed Consent

A majority of the participants emphasized the need for adequate information regarding the purpose and methods of the study, control of the biological samples after collection, and the credibility of the investigators. Participants voiced the need to both know and understand why their biological samples were needed for the intended research and what would be done with their samples in the context of the study.

“What would they (the researchers) do with it (the biological sample)? What kind of experiment would they do?”

“Depends on who was doing it (the research)-like some people like to do it for no reason. I need a real good reason.”

The reason for the research should also be transparent and of value to the participant. Without a valid reason for the research, collecting biological samples may be viewed with suspicion. In addition to informed consent, the participants suggested that tribal leaders provide their constituents with research-related information on the study that would be needed to make an informed decision. Information would be made available at tribal offices, community centers, and local stores for participants, and the community would be able to access and review the information on a continuous basis.

### 3.2. Traditional Beliefs

Traditional beliefs about the personal value of biological samples were described as having been passed down from parents and grandparents. The participants felt strongly about adhering to these beliefs in the conduct of their lives.

“It comes down from my grandpa’s point of thinking that I came here as a whole being. And that’s the way I want to go. I don’t want to be torn apart and go back home empty. In my family, we believe that…”

The participants further described their own experiences and upbringing with regard to various biological samples. The participants described the relative ease of giving blood and urine samples as part of routine health examinations. This was done with the understanding that the sample would be destroyed in the process and that test results would have direct and timely health benefit to the donor. In contrast, giving biological samples as a part of research, especially hair and nails, was viewed with caution. This perspective was expressed by a majority of the participants.

“I am willing to give blood or anything else like urine (in a medical clinic). It was the way I was raised, something about my grandma’s era and my mom’s-they don’t like to give (hair or nail) samples. It (this belief) was passed down to my mom.”

“I wouldn’t give anyone mine (hair and nails) to be studied.”

These responses highlight the differences between traditional attitudes, which view biological samples as an integral part of their being, with the perspectives of western research, in which biological samples are considered of more scientific than personal value.

### 3.3. Personal Choice

The decision to provide a biological sample for research purposes was also expressed by some participants as an individual choice that balanced traditional beliefs with the desire to contribute to the explanations for common metals-related diseases in the tribal community.

“We have all these spiritual aspects to our culture and… various things that we believe in. So about [biological samples]–we can work around it so it’s mostly an individual choice.”

Some of the participants indicated they would be inclined to provide biological samples under certain circumstances. These individuals would gain satisfaction from contributing to knowledge regarding the ill effects posed by metals exposures to themselves and/or other members of the community.

“I’d do it to see if it’s causing illness among the grownups (adults and elders). They may think it’s another illness rather than what it is.”

They also expressed their desire to contribute to the reduction of health risks. Their participation would also depend on whether they were adequately convinced of the validity of the objectives of a given study.

“Give them (potential research participants) really hard information on the consequences or risks to their health of the materials we use. To have (this) study benefits a lot of people. To know what precautions to take in the future with metals, would teach people what precautions to use.”

## 4. Discussion

The focus group responses reinforce the thesis that traditional perspectives play a central role when the Zuni decide to participate in research involving biological samples for environmental exposure assessment. Decision-making requires a balance between the perceived value of the proposed project and its purpose, with cultural perspectives surrounding the specific biological sample requested. Without recognizing this balance, researchers may incur risks to study participants in the form of unintended stress, anxiety or participation bias if the research is not conducted with respect for the traditional perspectives of the community. This discussion includes recommendations to aid native communities and researchers identify and avoid unintended risks when collaborating in environmental health research that proposes the use of biological samples for exposure assessment.

The focus group participants expressed a perspective of themselves as an integral whole. A feature of this traditional belief system is that the body is viewed as an integral whole; parts removed from the body may damage the spirit if not properly handled and/or destroyed [18,19]. This perspective highlights an inherent difference between traditional perspectives and Western research perspectives in which biological samples have greater scientific than personal value. Among the focus group participants, providing biological samples for medical diagnosis or treatment was considered more acceptable than providing biological samples for research. This is because diagnostic samples are collected for a specific purpose, discarded after analysis, and the results are returned in a timely manner with direct health-related benefit to the individual. In contrast, uncertainty about the purpose and use of biological samples in research, particularly hair and nail samples, raised concerns. Research which utilizes these samples may be inconsistent with the traditional norms expressed by participants. Therefore, although Western research may view blood samples as more invasive than hair and nail clippings, to the participants of this study, these samples were considered more invasive according to their traditional perspective. To avoid unintended suspicion, anxiety, or other consequences resulting from this difference in perspective, research involving any biological sample should be initiated with a transparent, community-specific evaluation of the research risks and benefits conducted in collaboration with the tribal community [15]. The process may include agreements to co-monitor study procedures and sample repositories in addition to providing post-research results to the participants and the community in a timely manner with an easily understood interpretation of the significance of the research [16,20].

Our focus group participants expressed the need to know details about the methods of research, a valid motivation behind the research, and the credibility of the researcher. These issues are summarized but not detailed in the consent process. Although non-Native American review boards and researchers may view such research-related risks as minor, the same risks may be viewed by Native participants as substantial. Native American communities are requesting a more extensive process for the disclosure of information than has been typically provided in the consent and results dissemination processes hitherto [21,22]. Native American communities have recently increased their use of independent ethics review panels and institutional review boards (IRB) to evaluate the impact of research on their communities, including potential conflicts between research objectives and traditional values, thus ensuring that proposed research questions, design, and methods are culturally appropriate [17,20,23,24,25].

The conflict among personal perspectives, traditional beliefs, and research methods can introduce several forms of research bias, which may be difficult to ascertain and control; however, such biases may have a profound influence on the results of a study. For example, eligible community members may decline to participate due to discomfort with either the goals or methodology of a study, or a combination of both, leading to participation bias. If research staff are members of the study community and therefore understand and identify with the community perspective, they may be uncomfortable violating their beliefs surrounding the collection of biological samples. In this instance, outside staff may be needed, which is often a preferable practice in small, tight-knit communities [26]. However, the use of external research staff, particularly for recruitment and interviewing, may produce additional bias. Given the potential harm that may stem from mishandling samples, the risk to the study community is increased with the maintenance of a biological sample repository. If not recognized and adequately addressed, the overall impact of these factors may not only bias study results but may influence the prospects of future research in native communities.

The challenges and recommendations to assure ethical conduct in community-based research have been well documented [15,27,28]. Sexton et al. (2015) suggested methods to engage with tribal IRBs to cultivate reflective, context-based research ethics that better consider the needs and concerns of communities in health studies involving the use of biological samples [13]. A common recommendation across these sources is for community-based researchers to maintain close communication with the community throughout the research process [14,15,25]. Additionally, the communication can extend to the maintenance of sample repositories as well as the fate of the biological samples collected for research.

### Limitations

We did not attempt to identify the reasons for non-participation among the individuals who were recruited but did not attend the focus group. However, given the wide age range and equal gender distribution of our participants, we do not anticipate bias in our focus group results. Participation in our study was restricted to members of a single Southwestern Native American community. Although additional study is needed to confirm the generalizability of our findings, the consistency of the three major themes identified throughout our focus group discussions as well as the consistency of these themes with previously reported tribal concerns suggests our findings are valid and generalizable.

## 5. Conclusions

The results of the focus group highlight the importance of conducting community-directed participatory research to develop best practices for ethical collection and use of biological samples in environmental health research. Failure to account for traditional beliefs at each step of the research process may incur unintended risks to the individual study participant and the tribal community; it may potentially bias the results of the study as well as damage trust, hindering future research opportunities in native communities.

## References

[1] US Environmental Protection Agency (2014). A Decade of Tribal Environmental Health Research: Results and Impacts from EPA’s Extramural Grants and Fellowship Programs.

[2] Lewis J., Gonzales M., Lewis J., Gonzales M., Burnette C., Benally N., Seanez P., Nez H., Nez C., Nez S. (2015). Environmental contaminants and potential risks for children in Native communities. J. Soc. Work Disabil. Rehabil..

[3] Harmon M., Lewis J., Miller C., Hoover J., Ali A., Shuey C., Cajero M., Lucas G., Pacheco B., Erdei E. (2018). Arsenic Contribution to Circulating Oxidized Low-density Lipoprotein in a Native Community. J. Toxicol. Environ. Health Part A.

[4] Harmon M.E., Lewis J., Miller C., Hoover J., Ali A.S., Shuey C., Cajero M., Lucas S., Zychowski K., Pacheco B. (2017). Residential proximity to abandoned uranium mines and serum inflammatory potential in chronically exposed Navajo communities. J. Expo. Sci. Environ. Epidemiol..

[5] Gonzales M., Erdei E., Hoover J., Nash J. (2018). A review of environmental epidemiology studies in southwestern and mountain west rural minority populations. Curr. Epidemiol. Rep..

[6] Newman J.D., Navas-Acien A., Kuo C.-C., Guallar E., Howard B.V., Fabsitz R.R., Devereux R.B., Umans J.G., Francesconi K.A., Goessler W. (2016). Peripheral Arterial Disease and Its Association With Arsenic Exposure and Metabolism in the Strong Heart Study. Am. J. Epidemiol..

[7] Mateen F.J., Grau-Perez M., Pollak J.S., Moon K.A., Howard B.V., Umans J.G., Best L.G., Francesconi K.A., Goessler W., Crainiceanu C. (2017). Chronic arsenic exposure and risk of carotid artery disease: The Strong Heart Study. Environ. Res..

[8] Kuo C.C., Howard B.V., Umans J.G., Gribble M.O., Best L.G., Francesconi K.A., Goessler W., Lee E., Guallar E., Navas-Acien A. (2015). Arsenic Exposure, Arsenic Metabolism, and Incident Diabetes in the Strong Heart Study. Diabetes Care.

[9] Zheng L.Y., Umans J.G., Yeh F., Francesconi K.A., Goessler W., Silbergeld E.K., Bandeen-Roche K., Guallar E., Howard B.V., Weaver V.M. (2015). The association of urine arsenic with prevalent and incident chronic kidney disease: Evidence from the Strong Heart Study. Epidemiology.

[10] Tellez-Plaza M., Guallar E., Howard B.V., Umans J.G., Francesconi K.A., Goessler W., Silbergeld E.K., Devereux R.B., Navas-Acien A. (2013). Cadmium Exposure and Incident Cardiovascular Disease. Epidemiology.

[11] Pausentbach D., Galbraith D. (2006). Biomonitoring: Is body burden relevant to public health?. Regul. Toxicol. Pharmacol..

[12] Metcalf S.W., Orloff K.G. (2004). Biomarkers of exposure in community settings. J. Toxicol. Environ. Health Part A.

[13] Saxton D.I., Brown P., Seguinot-Medina S., Eckstien L., Carpenter D.O., Miller P., Waghiyi V. (2015). Environmental health and justice and the right to research: Institutional review board denials of community-based chemical biomonitoring of breast milk. Environ. Health.

[14] Williams R., Willging C.E., Quintero G., Kalishman S., Sussman A.L., Freeman W.L., on behalf of RIOS Net Members (2010). Ethics of health research in communities: Perspectives from the southwestern United States. Ann. Fam. Med..

[15] Sterling R.L. (2011). Genetic research among the Havasupai: A cautionary tale. Virtual Mentor.

[16] National Research Council (2006). Human Biomonitoring for Environmental Chemicals.

[17] Mason S.M., Garroutte E., Turner Goins R., Nez Henderson P. (2004). Access, relevance and control in the research process: Lessons from Indian Country. J. Aging Health.

[18] Alvord L.A., Van Pelt E. (1999). The Scalpel and the Silver Bear: The First Navajo Woman Surgeon Combines Western Medicine and Traditional Healing.

[19] Romero F. (2001). American Indian and Alaska Native Genetics Research Policy Formulation Meeting: A Summary Meeting Report. Segment Three: Review Board’s Perspectives Regarding Genetics Research. https://www.nigms.nih.gov/News/reports/Documents/grpf_report.pdf.

[20] Isreal B.A., Schultz A.J., Parker A.E., Becker A.B. (1998). Review of community-based research: Assessing partnership approaches to improve public health. Ann. Rev. Public Health.

[21] Sharp R.R., Foster M.W. (2000). Involving study populations in the review of genetic research. J. Law Med. Ethics.

[22] Hiratsuka V.Y., Brown J.K., Hoeft J., Dillard D.A. (2012). Alaska Native people’s perceptions, understandings, and expectations for research involving biological specimens. Int. J. Circumpolar Health.

[23] Angal J., Petersen J.M., Tobacco D., Elliott A.J. (2016). Prenatal alcohol in SIDS and Stillbirth Network. Ethics review for a multi-site project involving Tribal Nations in the Northern Plains. J. Empir. Res. Hum. Res. Ethics.

[24] Kelley A., Belcourt-Dittloff A., Belcourt C., Belcourt G. (2013). Research ethics and indigenous communities. Am. J. Public Health.

[25] Brugge D., Missaghian M. (2006). Protecting the Navajo People through tribal regulation of research. Sci. Eng. Ethics.

[26] Norton I.M., Mason S.M. (1996). Research in American Indian and Alaska Native Communities: Navigating the cultural universe of value and process. J. Consult. Clin. Psychol..

[27] Flicker S., Travers R., Guta A., McDonald S., Meagher A. (2007). Ethical dilemmas in community-based participatory research: Recommendations for institutional review boards. J. Urban Health.

[28] McGrath M.M., Fullilove R.E., Kaufman M.R., Wallace R., Fullilove M.T. (2009). The limits of collaboration: A qualitative study of community ethical review of environmental health research. Am. J. Public Health.

